# Supplementary material for: Preparing for the future: The changing demographic composition of hospital patients in Denmark between 2013 and 2050
Source: PLoS One. 2020 Sep 30;15(9):e0238912. doi: 10.1371/journal.pone.0238912 (PMC7526879; doi:10.1371/journal.pone.0238912)
Supplement: S1 Table — (DOCX) [file pone.0238912.s001.docx]

**Supplementary Table S1:** Overview of projection assumptions.

| Process | Parameters | (Sub-) Population | Value |
| --- | --- | --- | --- |
| Mortality | e(0) in 2059 | Men | 87.1 years |
|  | (Lee-Carter Method) | Women | 89.5 years |
|  |  |  |  |
| Fertility | Long-Term TFR | Danish Origin - Danish Citizenship | 1.91 |
|  |  | Danish Origin - foreign Citizenship | 1.91 |
|  |  | Immigrants non-Western countries - Danish citizenship: | 1.68 |
|  |  | Immigrants non-Western countries - foreign citizenship: | 1.97 |
|  |  | Immigrants Western countries - Danish citizenship: | 1.61 |
|  |  | Immigrants Western countries - foreign citizenship: | 1.77 |
|  |  | Descendants from non-Western countries - Danish citizenship: | 1.91 |
|  |  | Descendants from non-Western countries - foreign citizenship: | 1.91 |
|  |  | Descendants from Western countries - Danish citizenship: | 1.75 |
|  |  | Descendants from Western countries - foreign citizenship: | 1.75 |
|  |  |  |  |
|  | Origin at birth | Danish origin - Danish citizenship | 100.00 |
|  | (frequencies of change) | Danish origin - foreign citizenship | 100.00 |
|  |  | Immigrants from non-Western countries - Danish citizenship | 24.50 |
|  |  | Immigrants from non-Western countries - foreign citizenship | 21.80 |
|  |  | Immigrants from Western countries - Danish citizenship | 73.10 |
|  |  | Immigrants from Western countries - foreign citizenship | 36.40 |
|  |  | Descendants from non-Western countries - Danish citizenship | 100.00 |
|  |  | Descendants from non-Western countries - foreign citizenship | 41.40 |
|  |  | Descendants from Western countries - Danish citizenship | 100.00 |
|  |  | Descendants from Western countries - foreign citizenship | 66.30 |
|  |  |  |  |
| Out-Migration | Number of out-migrants | entire Danish population and all groups of origin | N/A, not |
|  |  |  | further specified |
|  |  |  | as 2015-2017 levels |
|  |  |  |  |
| In-Migration | Number of in-migrants | Immigrants without Danish citizenship | 17,000 |
|  |  | Western immigrants without Danish citizenship | 28,100 |
|  |  | Re-immigration: Danish origin and all with Danish citizenship | N/A, not |
|  |  |  | further specified, |
|  |  |  | as 2015-2017 levels |
