# Supplementary material for: Preparing for the future: The changing demographic composition of hospital patients in Denmark between 2013 and 2050
Source: PLoS One. 2020 Sep 30;15(9):e0238912. doi: 10.1371/journal.pone.0238912 (PMC7526879; doi:10.1371/journal.pone.0238912)
Supplement: S2 Table — (PDF) [file pone.0238912.s002.pdf]

Supplementary Table S2: Comparing the impact of different specifications of hospital care use for 2013 (baseline year) and 2050 (last year of the projection period).

2013 - Men								
Age	(A) Days in hospital		(B) Days in hospital		(C) Days in hospital		(D) Days in hospital	
Group	N in Mio.	Share in %	N in Mio.	Share in %	N in Mio.	Share in %	N in Mio.	Share in %
0-14	0.20	4.32	0.20	4.16	0.30	3.82	0.30	3.68
15-49	0.54	11.70	0.54	11.25	0.93	12.02	0.94	11.63
50-69	0.82	17.54	0.82	16.87	1.29	16.61	1.29	15.98
70+	0.82	17.58	0.82	16.91	1.12	14.40	1.12	13.86
All	2.38	51.14	2.38	49.19	3.63	46.85	3.64	45.14
**2013 - Women**								
Age	(A) Days in hospital		(B) Days in hospital		(C) Days in hospital		(D) Days in hospital	
Group	N in Mio.	Share in %	N in Mio.	Share in %	N in Mio.	Share in %	N in Mio.	Share in %
0-14	0.17	3.61	0.17	3.47	0.25	3.27	0.25	3.15
15-49	0.52	11.24	0.71	14.63	1.10	14.15	1.40	17.32
50-69	0.66	14.10	0.66	13.56	1.47	18.98	1.47	18.27
70+	0.93	19.91	0.93	19.15	1.30	16.75	1.30	16.12
All	2.28	48.86	2.46	50.81	4.12	53.15	4.42	54.86
Total	4.66	100.00	4.84	100.00	7.75	100.00	8.05	100.00
**2050 - Men**								
Age	(A) Days in hospital		(B) Days in hospital		(C) Days in hospital		(D) Days in hospital	
Group	N in Mio.	Share in %	N in Mio.	Share in %	N in Mio.	Share in %	N in Mio.	Share in %
0-14	0.22	3.27	0.22	3.17	0.32	3.08	0.32	2.99
15-49	0.57	8.50	0.57	8.24	0.98	9.33	0.98	9.07
50-69	0.79	11.74	0.79	11.38	1.25	11.91	1.25	11.54
70+	1.94	28.89	1.94	28.01	2.57	24.56	2.57	23.78
All	3.52	52.40	3.52	50.81	5.12	48.89	5.12	47.38
**2050 - Women**								
Age	(A) Days in hospital		(B) Days in hospital		(C) Days in hospital		(D) Days in hospital	
Group	N in Mio.	Share in %	N in Mio.	Share in %	N in Mio.	Share in %	N in Mio.	Share in %
0-14	0.18	2.72	0.18	2.64	0.27	2.63	0.28	2.55
15-49	0.54	8.07	0.75	10.86	1.13	10.82	1.47	13.60
50-69	0.64	9.48	0.64	9.19	1.44	13.76	1.44	13.32
70+	1.84	27.33	1.84	26.50	2.50	23.90	2.50	23.15
All	3.20	47.60	3.41	49.19	5.35	51.11	5.69	52.62
Total	6.72	100.00	6.93	100.00	10.46	100.00	10.81	100.00
