# Supplementary material for: Preparing for the future: The changing demographic composition of hospital patients in Denmark between 2013 and 2050
Source: PLoS One. 2020 Sep 30;15(9):e0238912. doi: 10.1371/journal.pone.0238912 (PMC7526879; doi:10.1371/journal.pone.0238912)
Supplement: S1 Text — (DOCX) [file pone.0238912.s003.docx]

**Supplementary Text S1:** Further Remarks on sensitivity analyses.

Our main findings do not consider outpatient admissions and obstetrics-related admissions **(SETTING A)**. We investigated the impact of obstetrics-related admissions **(SETTING B)**. We speciﬁed this category as O.00 – O.99 and Z.30 – Z.39 according to the International Classiﬁcation of Diseases (ICD), 10th Revision. In addition, we estimated the contribution of those outpatient treatments which were provided within hospitals **(SETTING C)**. Each outpatient treatment was approximated to contribute exactly one day. We used this approximation for outpatient admissions since the registers provide neither the exact number of days an outpatient has spent in the hospital, nor an overview of the provided services. Instead, the information in the registers on outpatient treatments covers the start and the end date of the treatment period for administrative purposes. We further studied the combined impact of outpatient treatments and obstetrics-related admissions **(SETTING D)**.

While Supplementary Figure S1 compares age-and sex-specific trajectories for the baseline year 2014 reflecting these settings, Supplementary Figure S2 compares the results of the corresponding projections. A summary of projection results for all four settings is presented in Supplementary Table S2.
