# Supplementary figures and images for: Preparing for the future: The changing demographic composition of hospital patients in Denmark between 2013 and 2050
Source: PLoS One. 2020 Sep 30;15(9):e0238912. doi: 10.1371/journal.pone.0238912 (PMC7526879; doi:10.1371/journal.pone.0238912)

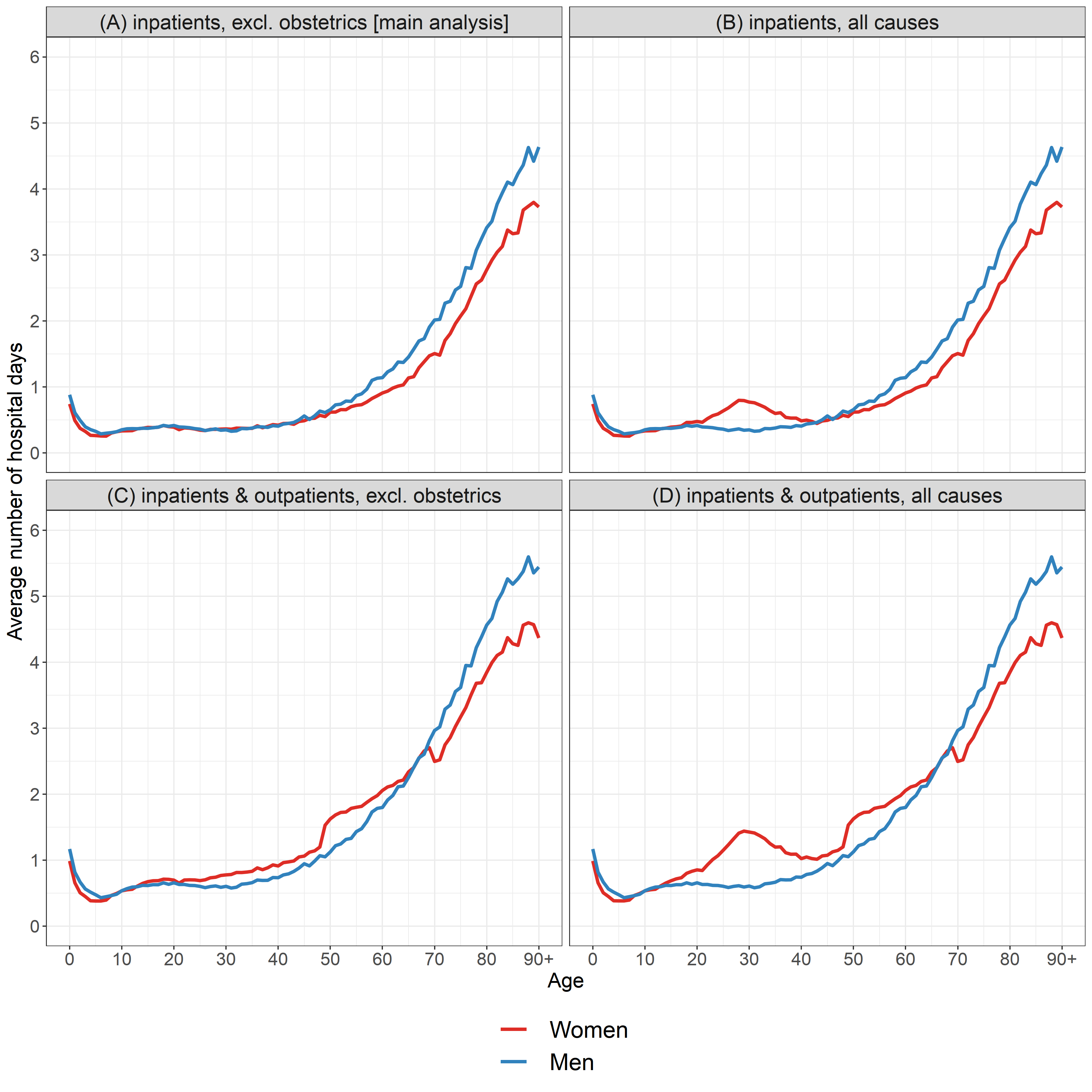

Supplement: S1 Fig — (TIF) [file pone.0238912.s004.tif]

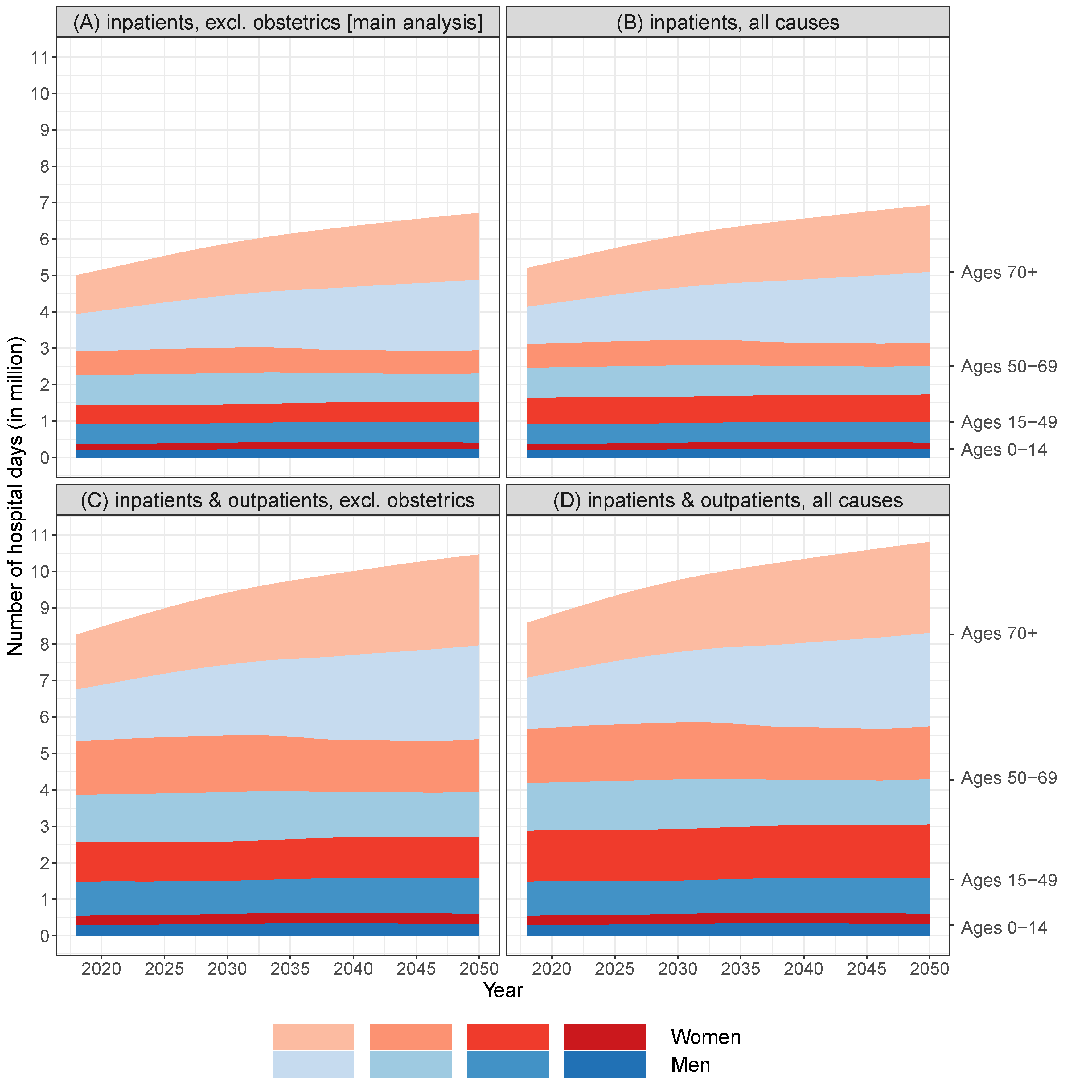

Supplement: S2 Fig — (TIF) [file pone.0238912.s005.tif]

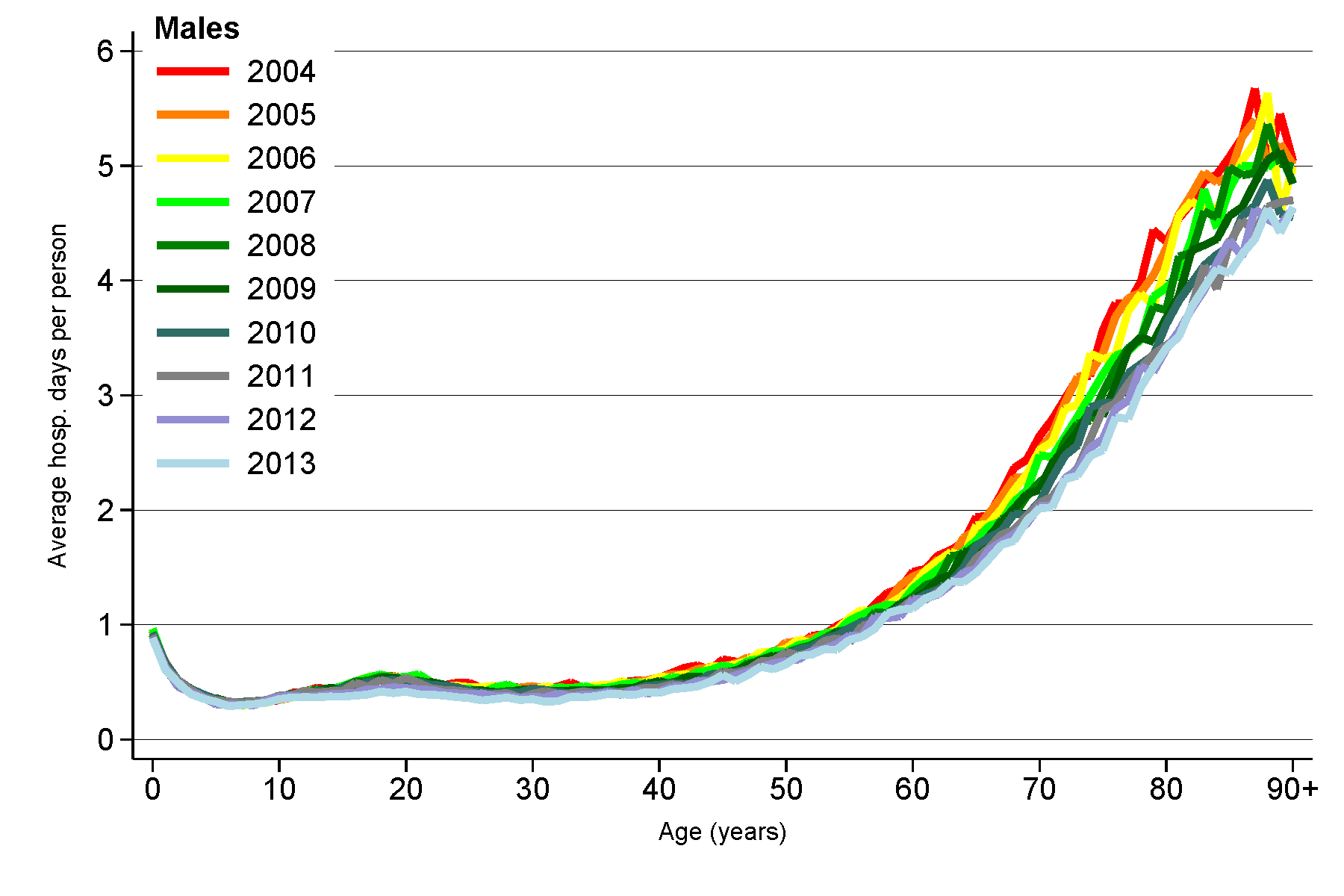

Supplement: S3 Fig — (TIF) [file pone.0238912.s006.tif]

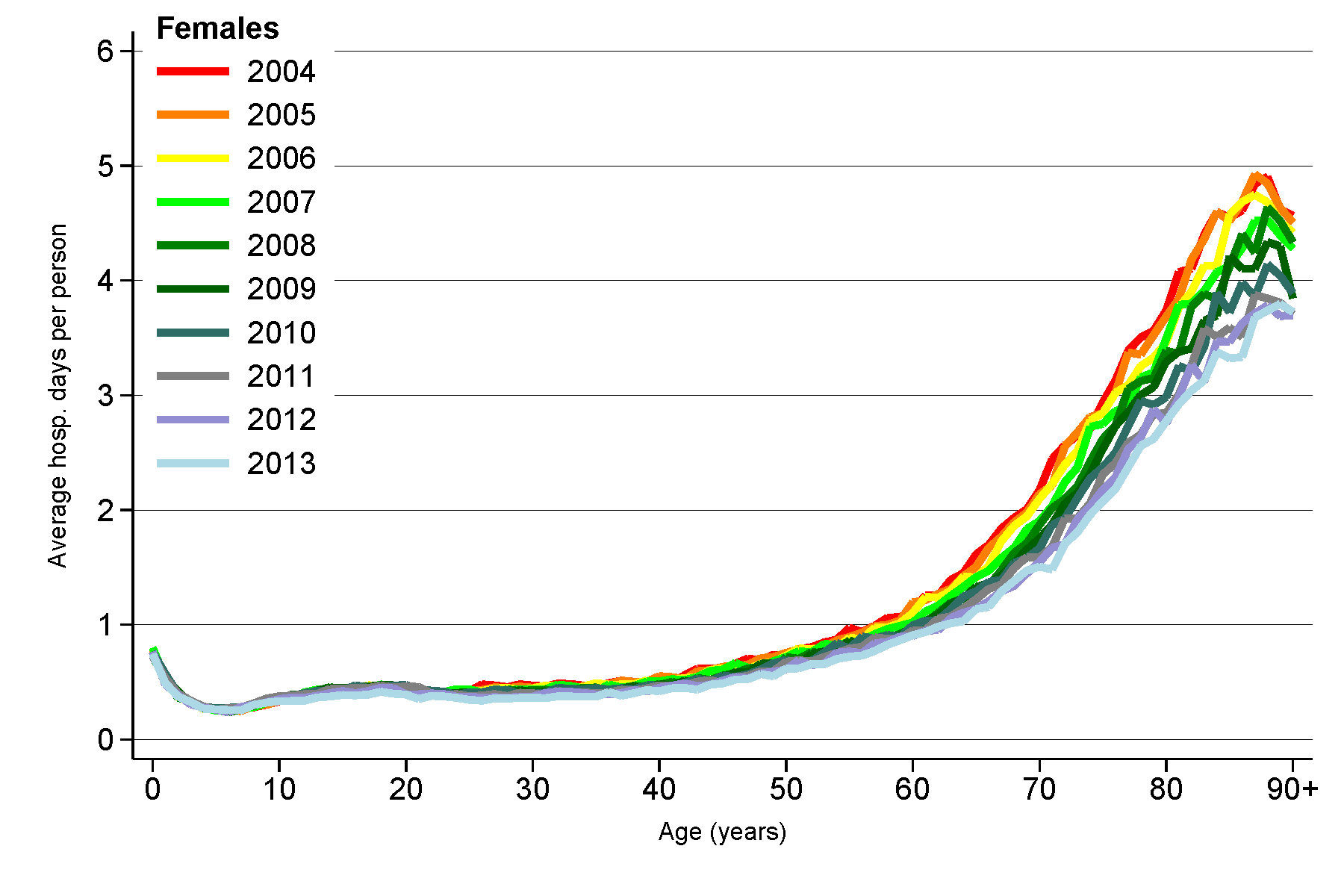

Supplement: S4 Fig — (TIF) [file pone.0238912.s007.tif]
